# Negative pressure pulmonary oedema after rhinoplasty

**DOI:** 10.4103/0019-5049.68393

**Published:** 2010

**Authors:** Rachna Wadhwa, Seema Kalra

**Affiliations:** Department of Anaesthesia, PGIMER and RML Hospital, New Delhi, India; 1 Department of Anaesthesiology and Critical Care, IGESI Hospital, Jhilmil, Delhi, India

Sir,

Ear, Nose and Throat (ENT) surgeries pose a challenge to the anaesthetists because of sharing of the same field and trickling. Postextubation problems are faced especially in nasal surgeries where both nares are packed and the patient is asked to breathe through the mouth. Often, the mandatory training for mouth breathing is not imparted by the ENT surgeons or the anaesthetist to the patients during the pre-anaesthetic check up.

An 18-year-old female, weighing 42 kgs, ASA status class 1, was scheduled for rhinoplasty. Laboratory investigations were within normal limits. The patient was counselled that her nose would be packed postoperatively and she would have to breathe through her mouth. The patient was premedicated with tablet alprazolam 0.25 mg, the night before operation.

General anaesthesia was induced with fentanyl 40 μg, propofol 80 mg and vecuronium 4 mg intravenously. The patient’s trachea was intubated with cuffed endotracheal tube of size 7.0 mm. Anaesthesia was maintained with O_2:_N_2_O (30:70), isoflurane and vecuronium bromide. Rhinoplasty progressed uneventfully and a plaster of paris cast was applied over the nose. The duration of surgery was 2.5 hours. Perioperatively, the patient received 1500 mL of Ringer lactate and her urine output was 300 mL. Neuromuscular blockade was reversed with neostigmine 2.5 mg and atropine 1.2 mg. The patient received diclofenac 50 mg intramuscularly for postoperative pain relief.

Postextubation, the patient became restless, tachypnoeic and started desaturating. 100% O _2_ delivery was tried with anatomical face mask but because of inadequate seal, the continuous positive airway pressure could not be achieved. So, the plaster of nose was removed. On auscultation, there were fine crepitations in most of the lung zones and there was drooling of pink frothy sputum from the angle of the mouth. As she was haemodynamically stable, pulmonary oedema was the obvious diagnosis. Furosemide 40 mg and morphine 6 mg were given intravenously. SpO_2_ improved to 94%. Portable chest radiograph was taken, which revealed bilateral fluffy shadows with normal cardiothoracic ratio. ECG and arterial blood gas analysis were normal. Gradually, the secretions decreased and SpO_2_ progressed to 100%. Head end of the bed was elevated and fluid restriction was implemented. The patient was observed for another hour in the operation theatre and was shifted to recovery room after that as she remained stable. Repeat roentgenogram was normal and she was discharged on the fifth postoperative day.

Negative pressure pulmonary oedema (NPPE) is noncardiogenic and is of two types. Type I is of sudden onset following upper airway obstruction and Type II develops after surgical relief of chronic upper airway obstruction. This is called NPPE because the laryngeal spasm, or other obstructive process in which the patient can inspire against the closed glottis (modified Müeller maneuver), is capable of generating an extremely negative intrapleural pressure (peaks of sustained inspiratory pressure between –50 cm H_2_O and –100 cm H_2_O, though the mean basal pressure is around –4 cm H_2_O)[1] which can trigger pulmonary oedema. The hydrostatic forces are the primary mechanism behind postobstructive pulmonary oedema and that the alveolar epithelium remains functionally intact in acute postobstructive pulmonary oedema.[2]

In Type II NPPE, it appears that the obstructive lesion produces a modest level of PEEP (positive end expiratory pressure) and increases end expiratory lung volume. Relief of the obstruction removes the PEEP and return lung volumes are preserved to normal. The sudden removal of PEEP leads to interstitial fluid transudation and pulmonary oedema.[3]

On radiological evaluation, NPPE is characterised by bilateral centralised pulmonary oedema, a wide vascular pedicle and a normal cardiothoracic ratio when the radiograph was obtained 15-165 min after the symptoms developed.[4]

To conclude, acute pulmonary oedema associated with obstruction of the upper airways can aggravate low morbidity surgeries, affecting mainly young patients. The knowledge of this complication and, most importantly, its prevention are crucial.
